# Supplementary material for: Role of Natural Products in the Management of COVID-19: A Saudi Arabian Perspective
Source: Healthcare (Basel). 2023 May 28;11(11):1584. doi: 10.3390/healthcare11111584 (PMC10252572; doi:10.3390/healthcare11111584)
Supplement: Supplementary file 1 [file healthcare-11-01584-s001.zip › healthcare-2361971-supplementary.pdf]

Role of Natural Products in the Management of COVID-19: A Saudi Arabian Perspective

Supplementary Table S1: Sociodemographic characteristics of the participants (n=677)

Characteristics	Variables	Frequency (Percentage)
Age	18-25 years	179 (26)
	26-40 years	336 (50)
	More than 40 years	162 (24)
Gender	Female	486 (72)
	Male	191 (28)
Nationality	Saudi	505 (75)
	Non-Saudi	172 (25)
Geographical location	Central region	371 (55)
	Eastern region	52 (08)
	Northern region	58 (09)
	Southern region	40 (06)
	Western region	156 (23)
Educational status	Intermediate school	28 (04)
	High school diploma	167 (25)
	Undergraduate	418 (62)
	Postgraduate degree	64 (10)
Employment status	Employed	365 (54)
	Not employed	220 (32)
	Student	92 (14)
Marital status	Married	383 (57)
	Single	263 (39)
	Separated	31 (05)
Family income	Less than 5000 SAR	145 (21)
	5000-10000 SAR	204 (30)
	More than 10000 SAR	328 (48)
Chronic diseases status	Yes	122 (18)
	No	555 (82)
Use of prescription drugs	Yes	118 (17)
	No	559 (83)
Commitment to MOH instruction for COVID-19	Full commitment	405 (60)
	Committed to some extent	259 (38)
	Not committed at all	13 (02)

Supplementary Table S2: Coefficients of five models of regression analysis

Coefficients[a]										
Model		Unstandardized Coefficients		Standardized Coefficients	t	Sig.	95.0% Confidence Interval for B		Collinearity Statistics	
		B	Std. Error	Beta			Lower Bound	Upper Bound	Tolerance	VIF
1	(Constant)	.501	.017		30.301	.000	.468	.533		
	NP use before COVID	.537	.013	.854	42.668	.000	.513	.562	1.000	1.000
2	(Constant)	.396	.024		16.515	.000	.349	.443		
	NP use before COVID	.516	.013	.821	40.395	.000	.491	.541	.924	1.083
	Priority for NP use always	.111	.019	.120	5.907	.000	.074	.147	.924	1.083
3	(Constant)	.349	.028		12.518	.000	.295	.404		
	NP use before COVID	.509	.013	.809	39.389	.000	.483	.534	.893	1.120
	Priority for NP use always	.104	.019	.113	5.583	.000	.068	.141	.914	1.094
	Combination of NP with other drugs COVID-19	.048	.015	.064	3.190	.001	.018	.078	.942	1.061
4	(Constant)	.314	.031		10.208	.000	.254	.375		
	NP use before COVID	.496	.014	.789	36.245	.000	.470	.523	.787	1.271
	Priority for NP use always	.098	.019	.106	5.215	.000	.061	.135	.898	1.113
	Combination of NP with other drugs COVID-19	.043	.015	.056	2.812	.005	.013	.072	.924	1.082
	NP use by family	.056	.021	.057	2.638	.009	.014	.098	.800	1.249
5	(Constant)	**.267**	.037		7.294	.000	.195	.339		
	NP use before COVID	**.497**	.014	.790	36.399	.000	.470	.524	.787	1.271
	Priority for NP use always	**.098**	.019	.106	5.243	.000	.061	.135	.898	1.113
	Combination of NP with other drugs COVID-19	**.041**	.015	.054	2.706	.007	.011	.071	.922	1.084
	NP use by family	**.059**	.021	.059	2.741	.006	.017	.100	.799	1.251
	Regions of Saudi Arabia	**.032**	.013	.046	2.393	.017	.006	.059	.996	1.004

a. Dependent Variable: NP for COVID

Supplementary Table S3: Impact of significant factors on the Use of NP for COVID-19

Model Summary[f]				
Model	R	R Square	Adjusted R Square	Std. Error of the Estimate
1	.854[a]	.730	**0.729**	.18090
2	.862[b]	.743	**0.742**	.17652
3	.864[c]	.747	**0.746**	.17533
4	.866[d]	.749	**0.748**	.17456
5	.867[e]	.751	**0.750**	.17395
a. Predictors: (Constant), NP use before COVID				
b. Predictors: (Constant), NP use before COVID, Priority for NP use always				
c. Predictors: (Constant), NP use before COVID, Priority for NP use always, Combination of NP with other drugs COVID-19				
d. Predictors: (Constant), NP use before COVID, Priority for NP use always, Combination of NP with other drugs COVID-19, NP use by family				
e. Predictors: (Constant), NP use before COVID, Priority for NP use always, Combination of NP with other drugs COVID-19, NP use by family, Regions of Saudi Arabia				
f. Dependent Variable: NP for COVID				
